# Developing and Evaluating an Interactive, Case-Based, Web-Based Active Learning Tool for Primary Care Physicians (Community Fracture Capture Learning Hub): Protocol for an Acceptability and Engagement Study

**DOI:** 10.2196/57511

**Published:** 2025-02-25

**Authors:** Ahmed M Fathalla, Cherie Chiang, Ralph Audehm, Alexandra Gorelik, Shanton Chang, Christopher J Yates, Steve Snow, Rahul Barmanray, Sarah Price, Lucy Collins, John D Wark

**Affiliations:** 1 Department of Medicine The Royal Melbourne Hospital, University of Melbourne Melbourne Australia; 2 Department of Diabetes and Endocrinology The Royal Melbourne Hospital, Melbourne Health Melbourne Australia; 3 Department of General Practice and Primary care, University of Melbourne Melbourne Australia; 4 School of Public Health and Preventive Medicine, Monash University Melbourne Australia; 5 School of Computing and Information Systems, University of Melbourne Melbourne Australia; 6 Praxhub Melbourne Australia; 7 Department of Obstetric Medicine Royal Women's Hospital, University of Melbourne Melbourne Australia; 8 Department of Endocrinology, Royal Children's Hospital Melbourne Australia

**Keywords:** community-based fracture capture bone hub, osteoporosis, virtual communities of practice, continuing professional development, primary care physicians, web-based learning platform, case-based education

## Abstract

**Background:**

The lack of osteoporosis treatment initiation after fragility fractures is a significant gap, especially in primary care. It is unclear whether barriers for primary care physicians (PCPs) arise from uncertainty about investigations, treatment initiation, or medication side effects. Key questions remain about whether active learning platforms improve treatment initiation rates better than passive methods and how PCP demographics affect learning outcomes. With PCPs increasingly using web-based platforms for continuing professional development due to time constraints and heavy workloads, an interactive community fracture capture (CFC) tool may serve as an effective alternative to in-person learning. Our CFC pilot study tested this new program’s design and content, showing promising potential.

**Objective:**

We aim to evaluate the interactive, case-based, web-based CFC Learning Hub, examining user acceptance and engagement with the platform, focusing on participants’ interactions, satisfaction levels, and overall experience.

**Methods:**

Participating PCPs are recruited through Praxhub, a web-based medical education platform, and provide electronic consent for data use after deidentification. They have been allocated into small groups (12-20 members) and join the CFC Learning Hub, a secure web-based community. This hub includes a web-based discussion forum with participant-contributed case studies and a knowledge repository. Over the 6-week program, participants will receive weekly modules with instructions, resources, discussion threads, and quizzes, along with interactive discussions moderated by experienced PCPs and physicians. The platform also hosts web-based surveys that, in combination with platform analytics, allow assessment of baseline knowledge gaps, level of activity or engagement, and improvements following the course completion. This study protocol demonstrates the creation and proposed evaluation of the CFC Learning Hub, featuring an interactive, case-based, small-group web-based learning platform equipped with flexibly scheduled, tailored modules to address the fracture treatment gap within the community. Both qualitative (via thematic analysis) and quantitative (by using 2-tailed paired *t* tests, Wilcoxon signed rank tests, and multivariable regression analysis) analyses will be used to assess levels of engagement and acceptance and changes in PCPs’ knowledge and confidence after engagement with the CFC Learning Hub.

**Results:**

Recruitment of participants started in May 2022. Data collection, analysis, and reporting will be completed following the completion of four 6-week cycles of the program.

**Conclusions:**

The study described in this protocol will provide important insights into the function and effectiveness of the CFC Learning Hub. This information will guide the expansion of the program. This initiative offers a simple digital solution for promoting current bone health practices tailored to PCPs’ needs and thereafter to expand the rollout of the e-learning hub and implementation of fracture liaison models at a primary care level in Australia and elsewhere. Future applications may extend to other clinical areas and professions.

**International Registered Report Identifier (IRRID):**

DERR1-10.2196/57511

## Introduction

### Osteoporosis: A Growing Global and National Concern and the Unmet Challenges in Fracture Prevention and Management

Osteoporosis affects around 200 million people globally, resulting in more than 8.9 million fractures each year, equivalent to 1 fracture every 3 seconds worldwide [1]. Furthermore, this disease burden is projected to increase dramatically in the coming decades as the global population is aging [2]. By the year 2050, the global occurrence of hip fractures is estimated to rise by 310% for men and 240% for women, compared with 1990 rates [3]. In Australia, osteoporosis incurred a societal cost of Aus $2.6 billion (~US $1.638 billion) in 2022 [2]. Despite the alarming increase in the prevalence of osteoporosis and the morbidity, mortality, and economic costs associated with low-trauma fractures in the aging population [4-6], there remains a substantial deficit in the implementation of effective interventions to prevent fragility fractures [7-9]. Despite the presence of effective diagnostic tools (eg, dual-energy x-ray absorptiometry [DXA]), fracture risk assessment tools (eg, FRAX) [10], and medications known to mitigate fracture risk, a substantial treatment gap exists in osteoporosis care, with only about 20% to 30% of individuals who sustain fragility fractures receiving guidelines-based care [2,11]. International and Australian data have unequivocally demonstrated the cost-effectiveness of hospital-based fracture liaison services (FLS) in secondary fracture prevention to address this treatment gap among patients presenting with low-trauma fractures, including in our own institution [12,13]. However, many patients with low-trauma fractures that may not require tertiary care (eg, vertebra, radius, and others) are investigated and managed purely at the primary care level and therefore may escape hospital-based services [14]. Furthermore, a recent survey of the International Osteoporosis Foundation Capture the Fracture program also noted a relative paucity of FLS centers in Australia [2]. The probability of initiation of osteoporosis medication following hip fracture within 12 months after discharge has been falling, despite these patients having the highest risk for imminent refracture [15,16]. The same investigation gap exists in primary fracture prevention among primary care physicians (PCPs); since 2016, there has been a plateau in subsidized bone density requests for Australians older than 70 years, despite the rapidly aging population [9,12,14,17].

### Adapting Fracture Prevention Models: The Urgent Need for Improved Primary Care Strategies

A rational approach to improving fracture prevention outcomes in the community setting would be to adapt the hospital-based model of care at the primary care level. However, unlike the hospital-based FLS that depends on the coordinator to facilitate investigations and clinic appointments, community-based fracture capture relies solely on PCPs to detect, investigate, and treat osteoporosis [18]. Despite compelling evidence for efficacious treatments to manage osteoporosis in primary care, and in particular the initiation of therapies to minimize the likelihood of refracture, the application of evidence and guidelines into clinical practice, particularly by PCPs, remains unacceptably low in Australia and many other countries such as the United States, Malaysia, Singapore, Germany, France, and Canada [9,19-24]. Consequently, many Australians are not receiving optimal, timely care in the community. For example, despite being eligible for osteoporosis-related investigations (eg, DXA) and antiresorptive therapies (eg, denosumab and bisphosphonates), a large proportion of Australians are neglected in this respect. While the hospital FLS model focuses on secondary fracture prevention with the motto of “Stopping at One Fracture,” the additional focus among PCPs should be primary fracture prevention with the aim of “Stopping the First Fracture” [25].

### Empowering PCPs: A Novel Approach to Bridging the Osteoporosis Treatment Gap Through Live, Interactive Learning Hubs

PCPs are faced with enormous challenges in daily practice to deal with multiple chronic health conditions, their sequelae, and comorbidities within a limited consultation time. Despite this, PCPs are required to continuously extend their knowledge to ensure that evidence-based practice is applied to their patients [26,27]. PCPs are increasingly seeking information on the web for their continuing professional development (CPD) [28-31], which is a viable alternative to face-to-face learning that potentially can be managed in their own time [32]. Hence, incorporating social media technologies for CPD has become a commonplace mechanism encouraging PCP learning in web-based group settings [29,33,34]. Including our Community Fracture Capture (CFC) Learning Hub pilot study, only a few recent studies have investigated the ability of web-based learning activities to mitigate the crisis in osteoporosis care by providing education, leveraging the scarce resources of the health care system, and enabling practitioners to offer better and more accessible skeletal health care locally and at a lower cost [35,36]. Our investigation of challenges PCPs face in using virtual communities of practice (VCoPs) for continuing medical education emphasizes the importance of trust building, efficient time management, and collaborative learning among health care professionals in building efficacious VCoPs [37]. We therefore aim to address the community osteoporosis treatment gap using a novel, live, and interactive CFC Learning Hub that provides a health VCoP to facilitate knowledge transfer to overcome barriers to osteoporosis recognition and management.

Although this program incorporates some elements typical of conventional courses, its distinctive combination of features and the unparalleled extent of flexible engagement opportunities set it apart. This uniqueness underscores our rationale for the study and the importance of conducting a formal research evaluation to fully assess its operational features, effectiveness, and potential impact on participants. Building on the feasibility study results, we aimed to create a more advanced version of the CFC Learning Hub with enhanced features. Therefore, this study aims to develop the CFC Learning Hub, an interactive web-based platform with case-based modules to improve fracture treatment among Australian PCPs and assess the impact of the CFC training model, while also examining the relationship between PCP demographics, clinical experiences, and knowledge gaps in treatment.

## Methods

### Study Context

The general design, development, and content planning for the interactive, patient-focused CFC Learning Hub have already been piloted to a small group of PCPs with favorable feedback and demonstrating excellent metric properties [35]. The CFC Learning Hub will be built from lessons learned from this feasibility study with the aim of enhancing PCPs’ awareness and competence in caring for patients with osteoporosis. The hub was conceived, designed, and developed by an interdisciplinary team of experts, comprising specialist physicians with expertise in osteoporosis and bone health, experienced PCPs, information technology and data science experts, and a project manager. It took time and resources for specialists to discuss and agree on major decisions for the program. Therefore, the timing and resource for such projects require appropriate support because of the need to understand terms, expectations, deliverables, and measurables across disciplines. The project team, situated in Melbourne, Australia, drew on its learning, teaching, and clinical experience to define the following important design criteria for PCP participants and elements in the project:

1. Practicing PCPs registered for the training have been provided with case studies and asked to provide their own case study with anonymized patients to encourage engagement.
2. There is interactive engagement between all parties involved.
3. Experienced PCP peers lead the group as facilitators, with guidance where needed by specialist “advisors,” promoting case discussions and guiding discussion of content according to its relevance and importance.
4. There is a minimum time commitment required for the award of CPD credits for participation.

Importantly, the CFC Learning Hub format is flexible in terms of the timing of participants’ input. Comments can be posted and discussions can be viewed at any time of the day, in order to accommodate participants’ workload and personal commitments. This learning platform provides an alternative to attending an on-site lecture or a webinar wherein a specific time slot needs to be set aside for attendance including discussion. The CFC Learning Hub will be driven by three principles: (1) sound learning pedagogy that is aimed at changing attitudes and behavior, supported by clear assessment structures; (2) learning analytics at the back end of the CFC Learning Hub to understand the development of the participants through studying their web-based participation in the discussion forum; and (3) usable and accessible features of the hub to ensure maximization of uptake of the system.

### Education Platform

Education has been delivered via Praxhub [38], an Australian-based web-based platform for health care professionals to access CPD resources from leading health care organizations. At the time of publishing this paper, Praxhub is used by thousands of health care professionals from more than 150 countries and aggregate education from more than 50 health care organizations. The CFC Learning Hub represents a departure from the main model of webinars and web-based learning modules for PCPs on a wider scale by including a range of increased opportunities for engagement and interactions with the participants.

The Praxhub platform allows for “groups” to be set up, in which participants can join and each week’s education modules can be hosted and made available to the participants. Before starting the CFC cycles, an early decision was made to make each of the groups “private”—meaning that only those participants invited into that group would have access to the modules and visibility of that group’s activity (ie, conversations within the discussion thread). It was considered important to quarantine the activity within the group from the remainder of Praxhub’s membership to create a more supportive and encouraging environment for individual participation. By extension, it was considered that a public group, in which conversations could be viewed by the broader Praxhub membership of health care professionals, may have the propensity to limit conversations (ie, active engagement) due to concern about criticism or professional judgement, as well as to avoid redundant discussions.

After assignment of participants to a designated private group, they attended an approximately 1-hour orientation session, conducted via a Zoom (Zoom Video Communications, Inc) meeting, to familiarize them with navigating the group and accessing the modules. Participants were informed by email (with a link in the email) of the availability of a new module each week. Within each module, participants were provided video or written instructions of the activities to be completed in that week, additional resources or materials to download and read, and a facilitated discussion thread in which to post questions to the specialists or PCP coordinator. The composition of each module is shown in Table 1. A weekly quiz was also made available for participants to probe their understanding of the week’s materials.

An additional consideration in selecting Praxhub as the system to deliver the CFC Learning Hub program was its robust data collection. While Praxhub strictly uses these data to enhance the user experience and report completions of CPD activities on the platform, in accordance with its privacy policy, participant consent was provided to Praxhub for the limited disclosure of their deidentified data for the purposes of this study (see the *Data Collection* and *Ethical Considerations* sections).

The research measured various user-system interaction points to allow us to quantify the participants’ engagement with the education program. These included dates and times (particularly time of day) when participants engaged with the education modules, whether videos were viewed or materials were downloaded, their engagement with other participants and the PCP coordinator and specialists via the discussion thread, and the responses to weekly quizzes.

These data were reviewed each week during the program to monitor participant-level engagement and at the end of each 6-week cycle to construct and understand how the participants engaged with the education and determine the performance in the program against the overall objectives.

**Table 1:** A summary of Community Fracture Capture Bone Hub 6-week content design.

Characteristic	Week 1	Week 2	Week 3	Week 4	Week 5	Week 6
Cases	Live webinar + 1× case	1× case	2× case	2× case	2× case	Live webinar + 1× case
Hot topics	Osteoporosis introduction and investigation	Assessment of risk factors	Osteoporosis effects	Treatment and side effects	Treatment failure	Difficult osteoporosis cases
Surveys	Premodule survey	N/A^a^	N/A	N/A	N/A	Postmodule survey
Quizzes	N/A	1×	1×	1×	1×	N/A

^a^N/A: not applicable.

### Education Program

The CFC Learning Hub is a web-based resource center for case studies, related resources to support PCP learning, and a web-based community discussion forum. Once enrolled, participants are required to watch a mandatory familiarization program to learn the function and capability of the hub and to submit 1 or 2 patient cases for discussion. Facilitators and specialist advisers will filter and select case studies that enable coverage of a syllabus of topics predetermined by members of the project team for this CPD course. Facilitators ensure that the posted case studies are anonymized and contain suitable, high-quality content for discussion. Each CFC Learning Hub cycle is scheduled for 6 weeks and comprises three main learning pillars: (1) osteoporosis investigation and treatment initiation, (2) treatment maintenance and monitoring, and (3) troubleshooting treatment side effects and treatment failure. In some cycles, a short video presentation will be delivered by a bone specialist to introduce the theme of that week. Participants will be exposed to a range of case studies over these modules and have access to resources to facilitate learning and novel paradigms of thinking about osteoporosis to address the treatment gap. In addition, there will be opportunities for peer-to-peer interactions and learning; the experience is enhanced and personalized by discussing difficulties that registrants encounter in their very own deidentified submitted cases. The CFC Learning Hub platform has two resources for PCP interactions: (1) a web-based knowledge repository (the “Knowledge Hub”) containing prepopulated evidence-based research papers, guidelines, position statements, and other resources relevant to that week’s topic curated by our team’s subject matter experts, and (2) a web-based social network forum (the “discussion forum”) where PCPs can freely post web-based comments including questions for discussion posted by facilitators; case studies as a way to encourage PCPs to learn and share their knowledge based on shared experiences and relevance to their immediate clinical practice; and topical discussions as either hot topics deemed relevant for PCPs, posted by our osteoporosis specialists, or other topics that were open for a wider discussion (ie, introductions (facilitators and PCPs introduce themselves) and burning questions (PCPs and facilitators post inquiries on osteoporosis; these will focus on significant issues arising during a group’s participation). The platform’s topical discussions section includes (1) diabetes and bone health, (2) atypical femoral fracture, (3) when to consider changing an osteoporosis therapy, (4) how to get the most from your patients’ bone density testing, (5) an “Introduction” topic for all users to introduce themselves, and (6) a facility for PCP participants to post inquiries based on seeking specific information about osteoporosis. Facilitators can also raise questions to promote discussion. These inquiries are organized under the term, “Burning Questions.” The Knowledge Hub will act as an accessible knowledge repository for all users at any time. It will be a dynamic source of information, being expanded and adapted in response to participants’ learning needs. Participating PCPs will complete 4 knowledge quizzes (1 quiz in each of weeks 2-5) and a questionnaire regarding osteoporosis caseload and demographics, confidence in case finding, investigations and treatment initiation, and barriers to osteoporosis case finding, investigations, and treatment initiation. The knowledge assessed will be in accordance with The Royal Australian College of General Practitioners (RACGP) and the Healthy Bones Australia (formerly Osteoporosis Australia) Osteoporosis Management and Fracture Prevention Guideline 2024 [39], a position statement developed by national experts to provide PCPs with clear guidance concerning the identification, investigation, and treatment of persons at risk for fragility fractures. In addition, pre- and postparticipation multiple-choice questions will be used for testing to assess knowledge transfer, change in confidence, and behavior (Table 1). The forum will be highly interactive (active learning) and case based and is supported by the RACGP and the Australian College of Rural and Remote Medicine approving the award of CPD points or hours to their fellows for active participation in the CFC Learning Hub (RACGP: 40 CPD points [2020-2022 triennium] and 12 activity hours [2023-2025 triennium], and Australian College of Rural and Remote Medicine: 8 Professional Development Program hours [February 23, 2020 framework] and 12 activity hours [2023-2025 framework]).

### Description of the Platform, Setting, Recruitment, and Participants

#### Platform

Praxhub has developed a web-based community (web and mobile apps) to support the delivery and distribution of medical education. It is free of charge for doctors to register and their credentials are validated against the government-held register of doctors. Praxhub already has a preexisting large network of registered PCPs in Australia and has been recruiting participants through their web page and internal member communication system.

The invitation to participate in the CFC Learning Hub will be distributed via PCPs professional bodies and using the existing pool of PCPs registered with Praxhub. Interested PCPs will contact the project manager, after which they will be screened for eligibility to participate in the program and subsequently enrolled if they satisfy the selection criteria and consent to participate in the project, approving to take part in the 6-week web-based educational program, answer pre- and postparticipation surveys, and allow the CFC Learning Hub to use their data for auditing and research purposes.

Once registered, all participants will be allocated to a private group, through which they can access the topical presentations, cases, and discussion forums. Participants and facilitators will have a profile, which shows their professional qualifications, experience, practice location, awards, and achievements, in accordance with the information that is required for the program, in addition to a profile picture (avatar) for personalization and easy identification. Participants will be able to engage in case studies and discussion topics and access and post comments, text, images, links to videos, and documents at their convenience.

The aim is to enroll 12-16 PCPs to participate per CFC Learning Hub cycle, and participants can drop in and out based on their interest in the content and their availability.

PCPs will be eligible to participate in the program if they (1) are a medical practitioner currently active in general practice in Australia, (2) submit at least 1 (preferably 2) suitable case studies with discussion or learning points from their own clinical experience that include patients at risk or those with prevalent osteoporosis, and (3) have web access.

PCPs will be excluded if they are unable to commit at least 6 hours to the anticipated time required to meet appropriate CPD guidelines.

#### Measures

Data related to sessions and activities of participants in the CFC Learning Hub will be collected for all cycles. Our primary focus, at this stage of the project, is detecting participation patterns to characterize the interactions of participating PCPs with the platform and specifically with respect to osteoporosis investigation and treatment initiation, learning, and knowledge-sharing behaviors related to participation and engagement in the CFC Learning Hub.

We will define the following terms from Praxhub analytics:

1. A session includes all user-related activities from initial logging in to logging out of the CFC Learning Hub. All activities made between the logging in and out activities will be grouped within a session.
2. An activity includes downloading of content, viewing posts and content, and posting comments.

Measuring sessions and activities will discover participants’ full active and passive engagement with the web-based system, which can be used to help measure the success of the platform (eg, high-session count means PCPs are frequently logging in, undertaking 1 or more activities, and then logging out). PCPs can actively engage through posting in a case study or topical discussion, while they can also engage passively via browsing and watching a brief video presentation in a case study or topical discussion. Initially, in the Knowledge Hub, we anticipate that PCP participation is likely mainly to be passive as PCPs browse the Knowledge Hub database. In addition, PCPs can actively engage with the Knowledge Hub by posting comments or queries. In measuring discussion forum use, the usage behavior for each case study and topical discussion will be assessed. For each case study and topical discussion, usage is measured from the creation date (on a Monday of each cycle’s week) until the end of the cycle).

#### Interactive Learning Hub

Once the 6-week program commences, the weekly content for 1 or more topics, along with a short video presentation on some topics (cycles 3 and 4), delivered by senior PCP and bone specialists to introduce the theme of that week, will be released by the moderators at a predefined scheduled time. This is communicated to participants via customized electronic correspondence allowing them to access and discuss. The content of these weekly modules is based on and selected from the following topics:

1. What is osteoporosis and why is it important?
2. Prevention strategies for osteoporosis and secondary fracture.
3. Who is at risk? Tools for assessing fracture risk.
4. Application in primary care.
5. The roles of allied health professionals—practice nurse, physiotherapist, and dietitian.
6. Physical activity and exercise: benefits and risks, beliefs about exercise.
7. Osteoporosis diagnosis and further evaluation. Appropriate initial workup.
8. DXA: Applications. What are its strengths and limitations?
9. Treatment: Approach to the choice of treatment and initiation of specific osteoporosis therapy.
10. Engaging patients in shared decision-making.
11. Calcium and vitamin D: Primary therapy and adjunctive therapy.
12. Monitoring for safety and effectiveness of therapy.
13. Recognizing and managing treatment “failure.”
14. Issues with long-term therapy and “treatment holidays”?
15. Osteonecrosis of the jaw and atypical fractures associated with treatment.
16. Why are patients being missed?

The hub will direct participants to resources and organizational websites in line with the week’s topic and case study. Along with the release of the educational resources, a relevant case study will be presented for discussion. Interactive and flexible discussion throughout the week will take place on predefined discussion boards within the CFC Learning Hub and will be moderated by PCP facilitators and experienced physicians.

#### Moderators and Data Gathering

Moderators will access the discussion boards during each topic discussion to monitor and participate in discussion at least once daily during each cycle. The time and way in which participants engage with the site will be quantitatively monitored throughout the duration of participation in the Learning Hub. Specifically, data that will be collected include (1) total number of discussion posts by each participant; (2) individual response time for each new discussion board opened on the web-based forum; (3) number of users who have actively participated in individual discussion board on the web-based forum; (4) the average time to reach a final resolution in each discussion on the web-based forum; (5) the accrued time that each participant spends logged onto the learning hub; (6) the total time that each participant spends on the web-based forum and individual pages on the learning hub, (7) the number of times internal and external websites are visited by each participant from the learning hub links; and (8) the overall popularity of web-based forums, as measured by number of page views, downloads, comments, rating, and followers of individual posts.

### Data Collection

#### Sample Size and Justification

The aim is to enroll 12-16 PCPs to participate per CFC Learning Hub cycle. A relatively small number of participants in a VCoP (ie, 12-20) who are learning and seeking knowledge is desirable as participants are likely more incentivized to be an active rather than passive user [40-44]. For example, the study by Barnett et al [41] reported that PCPs felt less isolated and more motivated to exchange knowledge after joining a small VCoP for their family physician training.

#### Qualitative Analysis

The qualitative analysis will aim to (1) identify common themes related to barriers and facilitators of web-based learning, PCP perceptions, and attitudes toward the program, and (2) examine barriers to effective detection and management of osteoporosis in general practices in Australia (ie, PCPs using a virtual space for their specific learning needs rather than physical seminars).

To achieve this, the relational content analysis will be undertaken [45,46]. More specifically, 2 researchers within the team will inductively identify codes independently in the first instance. In this initial step, researchers will use both semantic (capturing explicit and obvious meanings of the text) and latent (capturing tone, intention, and underlying assumptions) coding approaches to explore a wider range of codes. Subsequently, in the second phase, the 2 researchers will discuss and debate on the codes to assess for patterns that coalesce around key themes. The codes are then presented as subthemes of the themes that are identified. Finally, all data will then be counted against the list of subthemes to explore how strongly each subtheme is represented in the dataset by a single researcher and a random sample will be validated by a senior researcher.

Due to the innovative nature of the e-learning hub, themes will be created from participants’ transcriptions and validated through rigorously evaluating past and current initiatives made in this area (if any) in the literature. As the program progresses, we will seek to make necessary adjustments to the design and content of the e-learning hub.

The results of thematic analysis will be reported using a top to bottom approach with main (high level) themes reported first, following the subthemes emerged. All results will be supported by exemplary or representative quotes provided by participants.

We will also try to understand the differences in aforementioned areas based on PCPs’ years of experience and level of web-based activity (subject to diverse participation and data availability). Overall, feedback by participants will be used to reevaluate and redesign the e-learning hub in terms of navigating and searching for relevant content for PCPs learning needs. The qualitative analysis will aim to identify common themes related to barriers and facilitators of web-based learning, PCP perceptions, and attitudes toward the program. We will also try to understand the differences in aforementioned areas based on PCPs’ years of experience and level of web-based activity (subject to diverse participation and data availability). Due to the innovative nature of the e-learning hub, themes will be created from participants’ transcriptions and validated through rigorously evaluating past and current initiatives made in this area (if any) in the literature. As the program progresses, we will seek to make necessary adjustments to the design and content of the e-learning hub. Overall, feedback by participants will be used to reevaluate and redesign the e-learning hub in terms of navigating and searching for relevant content for PCPs’ learning needs.

#### Quantitative Analysis

The data from all cycles will be analyzed separately and combined. The between-cycle differences in participants demographic data (ie, years in practice) and level of activity, including time spent on the platform, number of various resources accessed during the training, and number of modules or education activities they have participated in, will be assessed using Kruskal-Wallis test. Changes in participants’ level of knowledge, measured using the proportion of correct responses provided to pre- and posteducation quizzes, will be assessed using either 2-tailed paired *t* tests or Wilcoxon signed rank tests, whereas multivariable regression analysis will be used to examine factors associated with levels of change in knowledge and confidence, while controlling for PCPs’ experience, level of participation in the program, and baseline level of knowledge or confidence. Other potential confounders will be considered, subject to the data availability and sufficient power. The level of significance for the quantitative analysis is set at α <.05.

### Ethical Considerations

The Melbourne Health Human Research ethics committee approved this project (site reference 2016.24), adhering to institutional ethical review processes for research involving human participants. Electronic consent from PCP participants who joined the CFC Learning Hub was obtained to use their deidentified data for research and auditing purposes. The electronic consent process included a waiver of consent for case study patients, whose anonymized information was also used in the project. The data collection and management of electronic consent were carried out using Praxhub tools, ensuring appropriate safeguards for privacy and confidentiality. The data used in this study were fully anonymized to protect participant identities. No compensation was provided to participants in this program.

## Results

Recruitment of participants started in May 2022 through initial contacts with PCP members on the Praxhub platform. The recruitment continued until August 2023. The expected quantitative and qualitative data on outcomes are currently being collected and their analysis will be available in early 2025.

## Discussion

### Overview

We hypothesize that (1) the CFC Learning Hub can provide a novel, peer-to-peer learning experience, offering up-to-date knowledge of primary and secondary fracture prevention strategies among a diverse range of PCPs, and (2) the improved confidence and motivation to investigate and treat osteoporosis, gained via interactive live web-based discussion among PCP peers and bone expert facilitators, can be quantified using predefined analytics tools.

International and Australian studies clearly establish the cost-effectiveness of hospital-based FLS for secondary fracture prevention, aiming to bridge the treatment gap for patients with low-trauma fractures [12,47]. Nevertheless, a considerable number of patients with low-trauma fractures receive investigation and management solely or predominantly from PCPs, potentially bypassing hospital-based services, or alternatively, they receive their ongoing care from their regular PCPs once acute fracture care is provided by hospital services [48]. Therefore, it is imperative for PCPs to maintain currency with fracture prevention protocols and primary care service delivery models. The translation and application of evidence and guidelines for osteoporosis management in primary care, particularly by PCPs, are unacceptably low internationally including in Australia, leading to underdiagnosis, underinvestigation, and undertreatment of osteoporosis in many countries [12,47,49-52]. Furthermore, a significant number of Australians are not receiving timely and appropriate management in primary care, as evidenced by the lack of access to osteoporosis-related investigations and therapies despite eligibility for such interventions [27,48,53]. An Australian review of 88,000 postmenopausal women attending PCPs found that 29% (20,248/69,358) of the targeted cohort had sustained at least 1 osteoporotic fracture, yet less than one-third of these women with fractures were receiving specific treatment for osteoporosis and 7% (3825/57,088) of these women were taking calcium alone [14]. Another Australian study reported that 76% (87/109) of men who had sustained an incident fracture remained untreated for osteoporosis 12 months after sustaining the fracture, despite being eligible for osteoporosis treatment [27]. These studies indicate that PCPs in Australia need additional resources to support primary care management of fragility fractures. Our education modules target the knowledge gaps specifically to address the issues of osteoporosis underdiagnosis and undertreatment and the need for timely and appropriate osteoporosis management.

Regarding the sufficiency of medical resources to cope with osteoporosis-related health issues, a recent paper presenting system dynamics modeling of the potential impact on fracture care of increasing hospital-based FLS demonstrated the probable inadequacy of these services to cope with the predicted fracture burden in years to come [54]. These observations underline the critical need to upgrade such care in the community, which inevitably will require high levels of PCP involvement. These analyses add emphasis to the need for effective education programs in this field.

### Comparison With Prior Work

PCPs encounter substantial daily challenges in providing optimal care for patients with various chronic health conditions, necessitating the application of evidence-based treatment approaches to enable the best outcomes [35,37]. Therefore, VCoPs for CPD may represent suitable alternatives for PCPs, being primary health care providers, to overcome barriers they encounter such as time constraints to accessing educational activities and a preference for face-to-face learning. Our collaborative group explored the challenges faced by PCPs in using VCoPs for their CPD, identifying seven themes crucial for designing and sustaining effective VCoPs: (1) low trust and risk perception, (2) patient information sharing, (3) timely responses, (4) relevant website access, (5) individual learning styles, (6) communication gaps between PCPs and specialists, and (7) the need for diversity and interactivity [37]. Following that exploratory study, few studies aimed to design virtual, interactive, and PCP-targeted bone health programs. In the United States, the Bone Health Tele Extension for Community Health Care Outcomes (TeleECHO) program [55] was proposed as one strategy that aimed at improving PCPs’ knowledge related to treatment gaps in osteoporosis care, using real-time videoconferencing to mentor health care professionals in rural and underserved communities [36]. In that study, 263 health care professionals from various countries participated over 21 months, showing substantial improvement in self-confidence across 20 domains of osteoporosis care with the TeleECHO intervention and addressing the osteoporosis treatment gap through enhanced education and knowledge-sharing among health care professionals. Quantitative and qualitative assessments were made of participating PCPs’ experience in our pilot program, which was developed because PCPs from different demographics might differ in their knowledge gap. Evidently, this CFC Learning Hub provided an innovative and interactive educational web-based tool to assist with increasing PCPs’ knowledge of osteoporosis management models and in turn the application of these guidelines to improve patient care [35]. The pilot program revealed the hot topics (eg, about osteoporosis, risk assessment of osteoporosis, and treatment and prevention strategies) that triggered the most discussion, indicating lack of confidence or knowledge gaps in these areas that are addressed in our current learning modules to ensure more widespread distribution of this knowledge. In our published pilot program of the CFC platform, PCPs were engaged in practice-based, web-based learning where they discussed their own cases with peer facilitators and specialist advisors [35]. In that program, our prototype CFC Learning Hub recruited 7 PCPs, as a convenience sample of PCPs interested in bone health topics, and it included 2 key components: a web-based discussion forum and a knowledge repository (the Knowledge Hub). The discussion forum contained anonymized case studies (contributed by PCP participants) and topical discussions (topics that were not case studies). Using 2 complementary tools (Google Analytics and Igloo Statistical Tool), we characterized individual participating PCPs’ engagement with the hub. The discussion included questions from participants directly to the facilitators regarding management dilemmas: this is a more practical tool to deliver knowledge than traditional questionnaires and quizzes. We measured the PCP participants’ behavior by quantifying the number of web-based sessions attended by the participants, activities undertaken within these web-based sessions, written posts made per learning topic, and their time spent per topic. We calculated time spent in both active and passive engagement for each topic. The baseline questionnaire highlighted the knowledge gaps and the facilitators, specifically directed discussion to address these gaps during the hot topic discussions. This project draws on findings from the pilot study and insights gained from implementing assessment tools to expand the CFC Learning Hub.

### Strengths and Limitations of the Program

Our educational program is positioned to deliver distinct outcomes compared with traditional face-to-face lectures and stand-alone, web-based webinars, offering PCPs time flexibility and leveraging the advantages of peer-to-peer and PCP-to-specialist interactions through web-based posts. It anticipates a departure from the traditional unidirectional nature of lectures by placing a strong emphasis on cultivating an interactive learning environment characterized by introspection and peer-to-peer discourse. This approach is designed to encourage participants to engage in more profound cognitive processing of the material, thereby fostering a nuanced comprehension. The integration of peer-to-peer discussions is a pivotal departure from conventional methods, establishing a collaborative and nonjudgmental forum for the exchange of insights, diverse perspectives, and shared learning experiences. This departure from traditional modes of education is anticipated to enhance the program’s efficacy, providing a more profound and enriching educational encounter for participants due to its multifaceted nature. Osteoporosis presents diverse challenges across age groups and between sexes, requiring tailored approaches in primary and secondary fracture prevention. The absence of a universal solution adds complexity, necessitating nuanced insights for specific cohorts, such as those undergoing oncological treatment, relying on glucocorticoids, or in the posttransplant phase. With various modalities such as pharmaceutical interventions, over-the-counter supplements, and exercise-based strategies, the program’s interactive and case-based nature is anticipated to align aptly with the intricate landscape of osteoporosis management, fostering a deeper understanding of this complex medical domain. For instance, our successful pilot study showcased the effectiveness of the program’s innovative approaches in tracing the path from engagement to practice change as recognized by obtaining CPD accreditation for the activity, thereby affirming the rationale for informing a larger study with a specific focus on creating and facilitating more practice-based topics for PCP participants [35].

The limitations of this study include transferability of this work beyond Australia since there is a shift toward community-based rather than hospital-based fracture prevention programs, and PCPs are encouraged to develop subspecialty interests in chronic condition such as osteoporosis. This in-depth subspecialty education might be beyond the scope of PCPs elsewhere in the world. The success of this approach is dependent on PCPs’ motivation to incorporate osteoporosis care into their workflow when it might be easier to refer to specialists. Personalized peer-to-peer discussion is more expensive to deliver per participant than traditional webinars and lectures and therefore might not be sustainable.

### Principal Findings

The main contribution of this protocol paper is a detailed description of how to conduct and analyze co-design, interactive, peer-to-peer PCP learning activities to develop a digital collaborative environment that promotes up-to-date bone health practice among PCPs. The program platform has capacity and agility to improve and adjust in real time based on participants number and needs.

The CFC Learning Hub will provide insights into the barriers to community-based osteoporosis identification and treatment initiation from the PCP perspective, as well as the effectiveness of a VCoP facilitated through an innovative web-based platform, along with methods for evaluating participation. The barriers identified might differ depending on region of practice and the experience level of the PCP. The pre- and postparticipation surveys permit quantification of knowledge and confidence gained. If successful, the CFC Learning Hub can be expanded to specifically target rural areas where interactive knowledge transfer and delivery can be logistically challenging.

### Conclusions

There is an urgent need to address the treatment gap in osteoporosis care, particularly within primary care settings where PCPs play a central role. The identified challenges, such as low trust of web-based content, patient information sharing, timely responses, and communication gaps, underscore the importance of tailored educational interventions such as the CFC Learning Hub. The pilot program’s success in engaging PCPs and addressing knowledge gaps suggests the potential of the CFC Learning Hub program and its innovative, web-based tools in improving osteoporosis management and patient care within primary care settings.

## Abbreviations

ACRRM: The Australian College of Rural and Remote Medicine
CFC: community fracture capture
CPD: continuing professional development
DXA: dual-energy x-ray absorptiometry
FLS: fracture liaison services
PCP: primary care physician
RACGP: The Royal Australian College of General Practitioners
TeleECHO: Tele Extension for Community Health Care Outcomes
VCoP: virtual community of practice

## Data Availability

The datasets generated during and analyzed during this study are available from the corresponding author on reasonable request, once the relevant findings have been published.
